# The Correlation of the Presence and Expression Levels of *cry* Genes with the Insecticidal Activities against *Plutella* *xylostella* for *Bacillus thuringiensis* Strains

**DOI:** 10.3390/toxins6082453

**Published:** 2014-08-19

**Authors:** Ming-Lun Chen, Pin-Hsin Chen, Jen-Chieh Pang, Chia-Wei Lin, Chin-Fa Hwang, Hau-Yang Tsen

**Affiliations:** 1 Department of Food Science, National Penghu University of Science and Technology, No. 300, Liuhe Rd., Magong City, Penghu County 880, Taiwan; E-Mail: vanco@gms.npu.edu.tw; 2 Department of Food Science and Technology, Hung Kuang University, No. 1018, Sec. 6, Taiwan Boulevard, Shalu District, Taichung City 43302, Taiwan; E-Mails: hkc5882247@hotmail.com (P.-H.C.); torico.lin@gmail.com (C.-W.L.); cfh1012@sunrise.hk.edu.tw (C.-F.H.); 3 Department of Biotechnology, Vanung University, No.1, Van-Nung Rd., Chung-Li, Tao-Yuan 32061, Taiwan; E-Mail: jcpang@mail.vnu.edu.tw

**Keywords:** *Bacillus thuringiensis*, insecticidal activity, PFGE, RAPD, *cry* genes

## Abstract

The use of *Bacillus* *thuringiensis* (*Bt*) strains with high insecticidal activity is essential for the preparation of bioinsecticide. In this study, for 60 *Bt* strains isolated in Taiwan, their genotypes and the correlation of some *cry* genes as well as the expression levels of *cry1* genes, with their insecticidal activities against *Plutella xylostella*, were investigated*.* Pulsed field gel electrophoresis (PFGE) and random amplified polymorphic DNA (RAPD) results revealed that the genotypes of these *Bt* strains are highly diversified. Also, a considerable number of the *Bt* strains isolated in Taiwan were found to have high insecticidal activities. Since strains that showed individual combined patterns of PFGE and RAPD exhibited distinct insecticidal activities against *P. xylostella*, thus, these genotypes may be useful for the identification of the new *Bt* strains and those which have been used in bioinsecticides. In addition, although the presence of *cry2Aa1* may have a greater effect on the insecticidal activity of *Bt* strains in bioassay than other *cry* genes, only high expression level of *cry1* genes plays a key role to determine the insecticidal activity of *Bt* strains. In conclusion, both RAPD and PFGE are effective in the differentiation of *Bt* strains. The presence of *cry2Aa1* and, especially, the expression level of *cry1* genes are useful for the prediction of the insecticidal activities of *Bt* strains against *P. xylostella*.

## 1. Introduction

*Bacillus thuringiensis* (*Bt*) is a gram positive, endospore forming bacterium of the *B. cereus* group that forms a parasporal crystal (δ-endotoxins or Cry toxins) during the stationary phase of its growth cycle [1]. The crystal consists of proteins exhibiting a highly specific insecticidal activity to a limited number of insect species [2,3]. Due to the insecticidal properties of *Bt*, products based on *Bt* toxins have been well recognized for the biological control of insect pests in forestry and agriculture for decades [4]. For *Bt*, the production of insecticidal crystal inclusion inside the cell during sporulation, is controlled by *cry* genes, which are usually located on the plasmid [5]. Many Cry toxins have been characterized and classified according to the nomenclature of Crickmore *et al.* [6]. Currently, more than 70 groups of Cry toxins have been identified [7]. Major classes of *Bt* toxins include Lepidoptera-active Cry1, Lepidoptera and Diptera-active Cry2, Coleoptera-active Cry3, and Diptera-active Cry4 [8].

Since *Bt* strains have been commonly used for the preparation of bioinsecticide, the isolation of novel *Bt* strains has become a routine activity [9]. The discrimination between new *Bt* isolates with insecticidal activity and those which have been used as bioinsecticides is also important from both ecological and economical views [10]. In this regard, the survey of the genotypes, the *cry* genes and the insecticidal activities of *Bt* strains may allow us to find novel *Bt* strains useful for industrial product preparation. Although the distribution of some *cry* genes in *Bt* isolates in Taiwan has been reported [11], little is known about their genotypes and the insect toxicity of these strains.

The diamondback moth, *Plutella xylostella* (Linnaeus) (Lepidoptera: Plutellidae), is the most destructive insect pest of cruciferous crops throughout the world. The characterization of toxicity of the *Bt* strains to the diamondback moth is important for agricultural purposes [12]. In this study, 60 *Bt* strains isolated from different origins including soil and granaries in Taiwan were characterized for their toxicity against *P. xylostella*. The genotypes of these strains were investigated by pulsed field gel electrophoresis (PFGE) and random amplified polymorphic DNA (RAPD), which have been proven to provide a high degree of discrimination for *Bt* strains [10,13]. Since the information on insecticidal activities of individual crystal proteins against selected arthropod has been reviewed by Frankenhuyzen [14], a collection of *cry* genes, including those coding for proteins biologically active to *P. xylostella*, were assayed based on polymerase chain reaction (PCR) for these strains. Furthermore, the expression level of *cry1* genes was determined; the concordance among insecticidal activity, PFGE and RAPD patterns, and the *cry* gene content as well as the expression level of *cry1* genes, was assessed.

## 2. Materials and Methods

### 2.1. Bacterial Strains and Cell Cultivation

*Bt* strains (TT1–TT62) used in this study were obtained from Taiwan Agriculture Chemical and Toxic Substances Research Institute (TACTRI), Taichung, Taiwan. These strains were mainly isolated from soil and granaries in Taiwan. Two strains, *i.e.*, TT12 and TT13, which were isolated from imported biopesticide products, were used as reference strains. Bacterial cells were cultivated in Brain Heart Infusion (BHI) broth (Difco™, Becton, Dickinson and Company, Sparks, MD, USA) overnight at 37 °C with rotary shaking (150 rev/min).

### 2.2. Bioassay of the Insecticidal Activity

The insecticidal activities of the *Bt* strains (TT1–TT62 except TT14) were assayed according to the methods described by Pang *et al.* [15]. *Bt* cells were cultivated, and the spores as well as crystal toxins were collected, mixed with Tris buffer (10 mM Tris-HCl, 10 mM EDTA, pH 7.4, Sigma-Aldrich, St. Louis, MO, USA), and assayed for protein concentration and insecticidal activity against *P. xylostella*. Two *Bt* strains, *i.e.*, TT12 and TT13, isolated from two commercial insecticide products, DiPel™ and XenTari™ (Abbott, Chicago, IL, USA), were used as positive control. Tris buffer was used as negative control. Protein concentration was assayed using bicinchoninic acid (BCA) protein assay kit (Pierce, Rockford, IL, USA). For each *Bt* strain, two protein doses were repeated 3 times using 10 larvae per assay. The spores and crystal mixtures at protein concentrations of 25 mg/L and 250 mg/L, were sprayed onto a 15 cm^2^ leaf piece of cabbage by a potter spray tower (Burkard Manufacturing Co. Ltd., Hertfordshire, UK) and the leaf were then fed to the third instar larvae at 25 °C under 60% humidity and incubated for 72 h. The insecticidal activities of the *Bt* strains were defined as mortality rates after incubation for 24, 48 and 72 h, which were calculated according to Abbott’s formula, *i.e.*, corrected mortality (%) = [(test mortality − blank control mortality)/(1 − blank control mortality)] × 100% [16]. All data were expressed as mean ± standard deviation (*n* = 3).

### 2.3. Genotyping by PFGE and RAPD

The isolation of chromosomal DNA of *Bt* strains (TT1–TT62 except TT23 and TT41) and *Not*I digestion were according to the methods described by Kolstø *et al.* [17]. Generally, bacterial cells harvested by centrifugation were washed, resuspended, and then mixed with low melting point agarose **(**NuSieve™ GTG™ agarose, FMC BioProducts, Rockland, ME, USA) to obtain the agarose plugs. Following the cell lysis and proteolysis, the restriction digestion was performed by placing a 2 mm slice of each plug into 100 μL of restriction buffer containing 20 U of *Not*I (New England Biolabs, Beverly, MA, USA). After incubation at 37 °C for 12–16 h, the plugs were placed into the slots of a 1.2% agarose gel in 0.5× TBE buffer (89 mmol/L Tris-borate, pH 8.3, 2 mmol/L EDTA, Sigma-Aldrich, St. Louis, MO, USA). Electrophoresis was performed by using CHEF-DR^®^II System (Bio-Rad, Hercules, CA, USA). The conditions used were 180 V for 28 h at 15 °C (pulse time: 7 to 90 s). Bacteriophage λ DNA concatemers (Bio-Rad, Hercules, CA, USA) were used as molecular weight markers.

For RAPD, bacterial genomic DNA was prepared by phenol-chloroform (Sigma-Aldrich, St. Louis, MO, USA) extraction [18], and then was subjected to RAPD reactions using a single 10-mer primer OPF-06 (5'-GGGAATTCGG-3') (MDBio, Inc., Taipei, Taiwan). RAPD reactions were performed in 25 µL reaction mixtures containing 1× PCR buffer (PROtech Technology Ent. Co., Ltd., Taipei, Taiwan), 300 µM of each deoxynucleoside triphosphate, 4 µM primer, 1.5 mM MgCl_2_ (PROtech Technology Ent. Co., Ltd., Taipei, Taiwan), 0.4 U of Prozyme (PROtech Technology Ent. Co., Taipei, Taiwan), and 50 ng of genomic DNA. RAPD conditions in a thermal cycler (Gene Amp PCR system 9600, Perkin Elmer, Norwalk, CT, USA) were as follows: 2 initial cycles consisted of 94 °C for 4 min, 35 °C for 2 min, and 72 °C for 2 min, followed by 40 cycles consisted of 94 °C for 20 s, 36 °C for 30 s, and 72 °C for 30 s, and a final extension at 72 °C for 7 min. The amplified products were analyzed by electrophoresis (Mupid-2 mini gel electrophoresis system, Cosmo Bio. Co. Ltd, Tokyo, Japan).

PFGE and RAPD patterns were analyzed by the NTSYSpc software (Numerical taxonomy and multivariate analysis system, version 2.10e, State University of New York, Stony Brook, NY, USA). Strains were clustered by using the Dice coefficient of similarity, and cluster analysis by unweighted pair group method with arithmetic averages (UPGMA). The final judgment of whether the patterns were identical was done by visual comparison.

### 2.4. Detection of cry Genes

Chromosomal DNA of each of the *Bt* strains was prepared by phenol-chloroform extraction [18]. All isolates were screened by PCR analysis for the presence or absence of 13 selected *cry* genes, including those coding for Cry proteins with insecticidal activity against *P. xylostella* [14]. The sequences of the primers were shown in Table 1. PCR amplifications were performed as previously described [19,20,21,22,23,24,25].

**Table 1:** Sequences of primers for *cry* genes and the sizes of amplified products.

Target genes	Primer sequences	Product size (bp)	Reference
*cry1*	F: 5'-CTGGATTTACAGGTGGGGATAT-3'	558	[19]
	R: 5'-TGAGTCGCTTCGCATATTTGACT-3'		
*cry1B*	F: 5'-CTTCATCACGATGGAGTAA-3'	367	[20]
	R: 5'-CATAATTTGGTCGTTCTGTT-3'		
*cry1E*	F: 5'-TAGGGATAAATGTAGTACAG-3'	1137	[21]
	R: 5'-MDATYTCTAKRTCTTGACTA-3'		
*cry2Aa1*	F: 5'-GTTATTCTTAATGCAGATGAATGGG-3'	498	[22]
	R: 5'-GAGATTAGTCGCCCCTATGAG-3'		
*cry3A*	F: 5'-CGTTATCGCAGAGAGATGACATTAAC-3'	951	[22]
	R: 5'-TGGTGCCCCGTCTAAACTGAGTGT-3'		
*cry4A2*	F: 5'-GGGTATGGCACTCAACCCCACTT-3'	1529	[22]
	R: 5'-GCGTGACATACCCATTTCCAGGTCC-3'		
*cry7*	F: 5'-CAACCAGACCTATTTTATTGGAGT-3'	476	[23]
	R: 5'-ATTTTTACAGCTGGAATTTTGTG-3'		
*cry8D*	F: 5'-AGAAACACAAGATAAAATACTCC-3'	401	[23]
	R: 5'-ATACAGCATCCCCTTCTACAATCT-3'		
*cry9A*	F: 5'-GGTTCACTTACATTGCCGGTTAGC-3'	1547	[24]
	R: 5'-GTTTGAGCCGCTTCACAGCAATCC-3'		
*cry9C*	F: 5'-CCACCAGATGAAAGTACCGGAAG-3'	1232	[24]
	R: 5'-GTTTGAGCCGCTTCACAGCAATCC-3'		
*cry9Ea*	F: 5'-GCGGCTGGCTTTACTTTACCGAG-3'	824	[24]
	R: 5'-GTTTGAGCCGCTTCACAGCAATCC-3'		
*cry22*	F: 5'-CAGATGAGATAGATGGGGATTTGA-3'	413	[23]
	R: 5'-ATTCGCTTCTATACTTGGCTGTC-3'		
*cry32Aa*	F: 5'-TGGTCGGGAGAGAATGGATGGA-3'	676–677	[25]
	R: 5'-ATGTTTGCGACACCATTTTC-3'		

### 2.5. Expression Levels of cry1 Genes

The expression levels of *cry1* genes in *Bt* strains were determined by a two-step reverse transcription real-time PCR. For each *Bt* strain, total RNA was prepared from 10^8^ CFU of *Bt* using PureLink™ RNA Mini Kit (Invitrogen Life Technologies, Carlsbad, CA, USA). Then, cDNA was synthesized in reactions containing 2 μL of total RNA from *Bt* isolates using a reverse transcription kit (SuperScript First-Strand Synthesis System, Invitrogen Life Technologies, Carlsbad, CA, USA). The cDNA was used as a template for real-time PCR to determine the expression level of *cry1* genes in *Bt* strains. Real-time PCR was performed in 20 µL reaction mixtures containing 1× KAPA FAST SYBR green I master mix (KAPA Biosystems, Woburn, MA, USA), 2 μL of the cDNA, and 0.25 mM of each primer to amplify all *cry1* genes. Real-time PCR was performed using the ABI 7500 system (Applied Biosystems, Foster, CA, USA). The amplification conditions in a thermocycler were according to the methods of Gaviria Rivera and Priest [26]. The expression levels of *cry1* genes were shown by threshold cycle (*Ct*) values.

### 2.6. Statistical Analysis

Statistical tests were performed using SPSS 17.0 (SPSS Inc., Chicago, IL, USA), *p* < 0.05 was taken as statistically significant. A paired samples *t*-test was applied to compare the mortality rates at different time points. Since multiple *cry* genes may be involved in the insecticidal activity of a *Bt* strain, a multiple regression analysis was applied to determine the relationship between the presence or absence of *cry* genes and the insecticidal activities of Bt strains. A Mann-Whitney *U* test was used to determine if the *Bt* strains with high expression levels of *cry1* genes showed higher insecticidal activities against *P. xylostella* than those with low expression levels of *cry1* genes.

## 3. Results

### 3.1. Insecticidal Activity of Bt Strains

**Figure 1:**
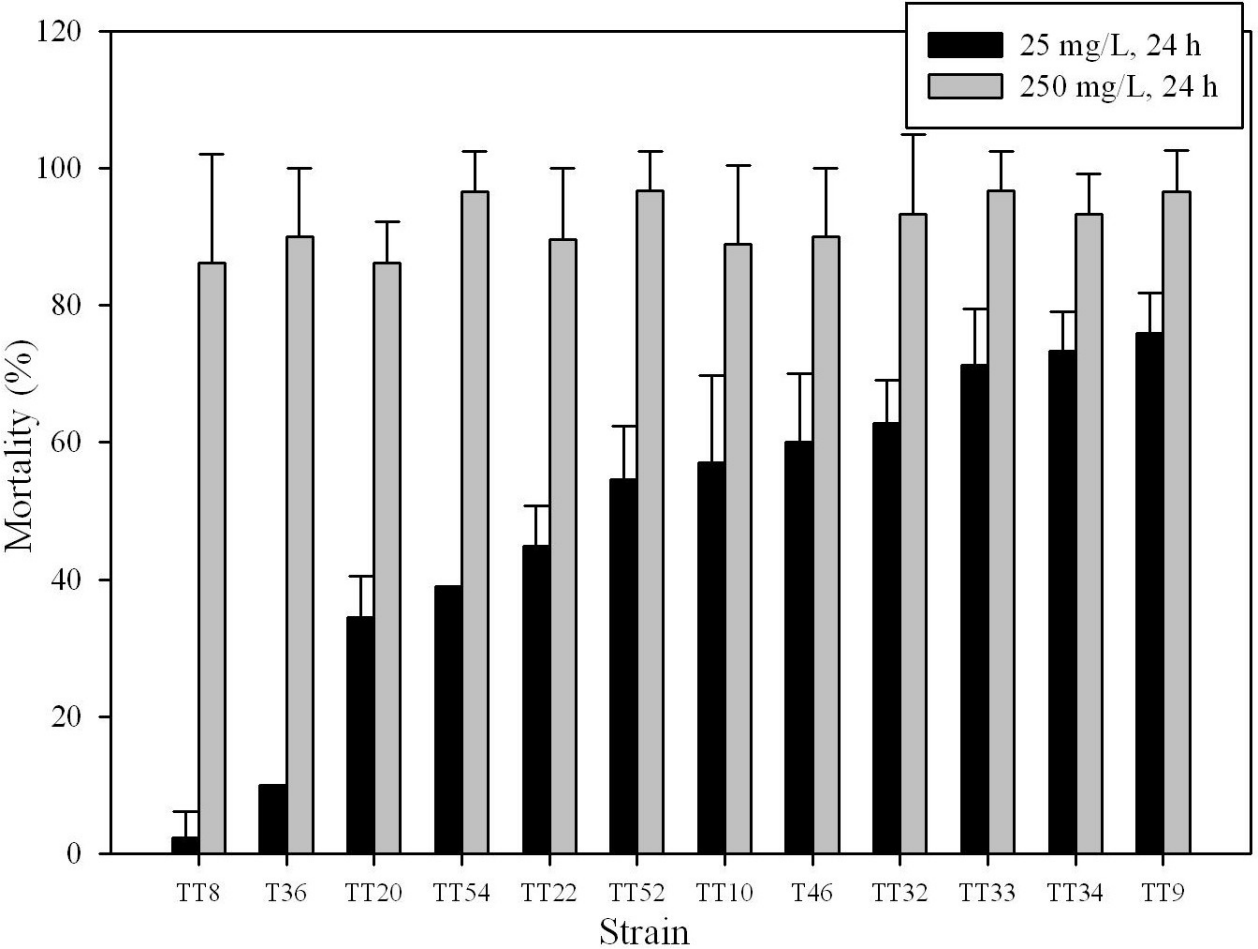
Part of the insecticidal activity of *Bt* strains assayed at two global Cry protein concentrations after 24 h incubation. Each vertical bar represents the mean ± standard deviation (*n* = 3).

To evaluate the insecticidal activity of *Bt* strains, and the effect of the dose of Cry proteins and the incubation time of larvae with these proteins, two concentrations of global Cry protein, *i.e.*, 25 mg/L and 250 mg/L, and three incubation time periods, *i.e.*, 24, 48, and 72 h, respectively, were used. Results were shown in Table 2. At the protein concentration of 250 mg/L, it was found that 35, 46, and 46 *Bt* strains showed mortality rates ≥90% after incubation for 24, 48, and 72 h, respectively. On the other hand, at the protein concentration of 25 mg/L, 11, 28, and 29 strains showed mortality rates ≥90% after incubation for 24, 48, and 72 h, respectively. At least, 19 strains showed higher insecticidal activities than those of the reference strains, *i.e.*, strains TT12 and TT13, at the toxin concentration of 25 mg/L, after incubation for 24 h (Table 1). A paired samples *t*-test was applied to compare the mortality rates at different time points. At the protein concentration of 25 mg/L, mortality rates after 72, and 48 h incubation were significantly higher than those after 24 as well as 48 h, and 24 h incubation, respectively (*p <* 0.05, degree of freedom (*df*) = 182). At the protein concentration of 250 mg/L, mortality rates after 48 h incubation were significantly higher than those after 24 h incubation (*p <* 0.05, *df* = 182); mortality rates after 72 h incubation were higher than those after 48 h incubation but not statistically significant. The above results indicate that the mortality of larvae gradually increases with the length of incubation time, and an assay at a protein concentration of 250 mg/L may not effectively differentiate the insecticidal activities of these *Bt* strains. Part of the results assayed at the global Cry protein concentration of 25 mg/L and 250 mg/L, respectively, with incubation time of 24 h, was shown in Figure 1. Thus, the insecticidal activity against *P. xylostella* at the global Cry protein concentration of 25 mg/L was used for further analysis.

**Table 2:** Comparison of the random amplified polymorphic DNA (RAPD) and pulsed field gel electrophoresis (PFGE) patterns, and insecticidal activity of *B. thuringiensis* strains.

Strain	RAPD pattern	PFGE pattern	Mortality (%) ^1^					
			250 mg/L			25 mg/L		
			24 h	48 h	72 h	24 h	48 h	72 h
TT1	D11	PT11	6.83 ± 0.00	12.57 ± 10.93	19.00 ± 12.00	4.24 ± 3.71	11.91 ± 1.14	10.19 ± 4.81
TT2	D12	PT12	6.90 ± 1.04	20.07 ± 7.28	22.72 ± 3.70	4.55 ± 3.94	5.28 ± 6.31	4.94 ± 4.28
TT3	D13	PT13	2.66 ± 4.60	1.49 ± 1.43	0.00 ± 0.00	0.00 ± 0.00	0.00 ± 0.00	0.00 ± 0.00
TT4	D14	PT14	0.00 ± 0.00	0.00 ± 0.00	12.67 ± 21.94	3.33 ± 5.77	2.33 ± 4.04	4.62 ± 4.00
TT5	D15	PT15	100.00 ± 0.00	100.00 ± 0.00	100.00 ± 0.00	96.55 ± 5.98	100.00 ± 0.00	100.00 ± 0.00
TT6	D16	PT16	93.10 ± 5.98	96.36 ± 6.31	96.00 ± 6.93	86.20 ± 15.81	100.00 ± 0.00	100.00 ± 0.00
TT7	D17	PT17	96.86 ± 5.44	96.68 ± 5.74	96.33 ± 6.35	86.20 ± 5.98	96.33 ± 6.35	100.00 ± 0.00
TT8	D18	PT18	86.20 ± 15.81	100.00 ± 0.00	100.00 ± 0.00	2.28 ± 3.94	16.33 ± 5.77	15.12 ± 6.68
TT9	D19	PT19	96.55 ± 5.98	100.00 ± 0.00	100.00 ± 0.00	75.85 ± 5.98	100.00 ± 0.00	100.00 ± 0.00
TT10	D20	PT20	88.89 ± 11.51	100.00 ± 0.00	100.00 ± 0.00	57.04 ± 12.75	89.33 ± 0.58	92.63 ± 6.40
TT11	D21	PT21	4.55 ± 3.95	30.78 ± 6.31	26.70 ± 6.68	2.28 ± 3.94	2.00 ± 0.00	0.00 ± 0.00
TT14	D24	PT23	ND ^2^	ND	ND	ND	ND	ND
TT15	D25	PT24	93.10 ± 11.95	96.36 ± 6.31	96.00 ± 6.93	14.91 ± 20.19	38.00 ± 45.40	42.13 ± 40.09
TT16	D26	PT25	30.99 ± 5.98	74.50 ± 12.62	76.85 ± 11.57	4.55 ± 3.94	5.67 ± 6.35	2.47 ± 4.28
TT17	D27	PT26	100.00 ± 0.00	100.00 ± 0.00	100.00 ± 0.00	79.30 ± 10.35	100.00 ± 0.00	100.00 ± 0.00
TT18	D28	PT27	96.17 ± 6.63	100.00 ± 0.00	100.00 ± 0.00	65.49 ± 33.28	96.33 ± 6.35	96.14 ± 6.68
TT19	D29	PT28	4.55 ± 3.94	1.64 ± 0.00	2.47 ± 4.28	4.93 ± 4.31	17.01 ± 5.74	24.54 ± 9.62
TT20	D30	PT29	86.20 ± 5.98	100.00 ± 0.00	100.00 ± 0.00	34.44 ± 5.98	74.33 ± 12.70	72.99 ± 13.36
TT21	D31	PT30	68.94 ± 0.00	92.71 ± 6.31	92.00 ± 6.93	17.18 ± 0.00	38.07 ± 6.31	45.99 ± 6.68
TT22	D32	PT31	89.65 ± 10.35	100.00 ± 0.00	100.00 ± 0.00	44.79 ± 5.98	92.67 ± 12.70	92.28 ± 13.36
TT23	ND	ND	3.33 ± 5.57	11.51 ± 14.37	20.67 ± 6.35	3.33 ± 5.77	10.07 ± 6.26	20.10 ± 6.57
TT24	D33	PT32	48.13 ± 3.23	89.66 ± 10.34	89.66 ± 10.34	3.33 ± 5.77	10.38 ± 5.97	13.82 ± 5.97
TT25	D34	PT33	100.00 ± 0.00	100.00 ± 0.00	100.00 ± 0.00	93.10 ± 11.95	96.33 ± 6.35	96.14 ± 6.68
TT26	D35	PT34	100.00 ± 0.00	100.00 ± 0.00	100.00 ± 0.00	93.33 ± 5.77	96.67 ± 5.77	96.49 ± 6.07
TT27	D36	PT35	100.00 ± 0.00	100.00 ± 0.00	100.00 ± 0.00	93.33 ± 5.77	100.00 ± 0.00	100.00 ± 0.00
TT28	D22	PT36	100.00 ± 0.00	100.00 ± 0.00	100.00 ± 0.00	89.27 ± 11.12	96.33 ± 6.35	96.11 ± 6.74
TT29	D34	PT37	100.00 ± 0.00	100.00 ± 0.00	100.00 ± 0.00	93.33 ± 5.77	100.00 ± 0.00	100.00 ± 0.00
TT30	D37	PT38	100.00 ± 0.00	100.00 ± 0.00	100.00 ± 0.00	93.33 ± 11.55	100.00 ± 0.00	100.00 ± 0.00
TT31	D38	PT39	96.67 ± 5.77	100.00 ± 0.00	100.00 ± 0.00	90.00 ± 17.32	96.67 ± 5.77	96.49 ± 6.07
TT32	D22	PT40	93.33 ± 11.55	100.00 ± 0.00	100.00 ± 0.00	62.77 ± 6.32	100.00 ± 0.00	100.00 ± 0.00
TT33	D39	PT41	96.67 ± 5.77	100.00 ± 0.00	100.00 ± 0.00	71.20 ± 8.27	90.33 ± 0.58	89.80 ± 0.55
TT34	D40	PT41	93.33 ± 5.77	100.00 ± 0.00	100.00 ± 0.00	73.33 ± 5.77	100.00 ± 0.00	100.00 ± 0.00
TT35	D38	PT42	63.33 ± 5.77	100.00 ± 0.00	100.00 ± 0.00	21.67 ± 2.89	77.67 ± 10.97	87.94 ± 13.01
TT36	D41	PT43	90.00 ± 10.00	100.00 ± 0.00	100.00 ± 0.00	10.00 ± 0.00	44.85 ± 5.97	58.63 ± 10.34
TT37	D42	PT44	80.00 ± 34.64	100.00 ± 0.00	100.00 ± 0.00	46.67 ± 5.77	96.67 ± 5.77	96.55 ± 5.97
TT38	D43	PT45	80.00 ± 26.46	100.00 ± 0.00	100.00 ± 0.00	33.33 ± 11.55	72.33 ± 11.55	72.42 ± 11.94
TT39	D44	PT46	96.67 ± 5.77	100.00 ± 0.00	100.00 ± 0.00	33.75 ± 5.30	76.73 ± 3.66	76.73 ± 3.66
TT40	D36	PT47	90.00 ± 17.32	100.00 ± 0.00	100.00 ± 0.00	50.00 ± 43.59	100.00 ± 0.00	100.00 ± 0.00
TT41	ND	ND	10.00 ± 10.00	17.00 ± 0.00	8.00 ± 6.93	6.67 ± 5.77	6.93 ± 0.00	0.00 ± 0.00
TT42	D34	PT48	96.67 ± 5.77	100.00 ± 0.00	100.00 ± 0.00	43.33 ± 5.77	86.33 ± 15.82	92.03 ± 6.91
TT43	D45	PT49	13.33 ± 5.77	37.95 ± 10.34	40.19 ± 11.96	0.00 ± 0.00	4.62 ± 4.00	2.87 ± 2.49
TT44	D46	PT50	40.00 ± 0.00	58.63 ± 10.34	52.00 ± 12.00	3.33 ± 5.77	20.72 ± 5.97	12.28 ± 6.91
TT45	D47	PT50	96.67 ± 5.77	100.00 ± 0.00	100.00 ± 0.00	83.33 ± 5.77	96.67 ± 5.77	96.01 ± 6.91
TT46	D33	PT51	90.00 ± 10.00	96.55 ± 5.87	96.00 ± 6.93	60.00 ± 10.00	79.33 ± 10.50	80.06 ± 13.81
TT47	D43	PT52	96.67 ± 5.77	100.00 ± 0.00	100.00 ± 0.00	74.53 ± 9.47	84.00 ± 10.39	83.42 ± 10.50
TT48	D43	PT52	93.33 ± 5.77	100.00 ± 0.00	100.00 ± 0.00	60.00 ± 10.00	82.67 ± 6.35	88.04 ± 11.96
TT49	D43	PT52	88.57 ± 2.48	100.00 ± 0.00	100.00 ± 0.00	80.00 ± 10.00	96.67 ± 5.77	96.01 ± 6.91
TT50	D43	PT52	100.00 ± 0.00	100.00 ± 0.00	100.00 ± 0.00	83.33 ± 15.28	100.00 ± 0.00	100.00 ± 0.00
TT51	D48	PT1	70.00 ± 10.00	96.55 ± 5.97	96.00 ± 6.93	3.33 ± 5.77	24.33 ± 6.35	24.24 ± 6.91
TT52	D38	PT53	96.67 ± 5.77	100.00 ± 0.00	100.00 ± 0.00	54.53 ± 7.85	84.33 ± 13.80	82.06 ± 15.82
TT53	D41	PT54	96.61 ± 5.87	100.00 ± 0.00	100.00 ± 0.00	86.45 ± 5.87	89.33 ± 10.50	88.91 ± 11.09
TT54	D2	PT2	96.61 ± 5.87	100.00 ± 0.00	100.00 ± 0.00	39.02 ± 0.00	47.42 ± 10.52	51.96 ± 6.40
TT55	D49	PT55	10.00 ± 10.00	17.27 ± 10.34	20.72 ± 5.97	3.33 ± 5.77	7.00 ± 0.00	13.82 ± 5.97
TT56	D50	PT56	0.00 ± 0.00	3.58 ± 3.10	0.00 ± 0.00	0.00 ± 0.00	0.00 ± 0.00	0.07 ± 0.13
TT57	D51	PT57	6.54 ± 5.80	4.45 ± 4.07	1.00 ± 1.73	2.85 ± 4.93	3.58 ± 3.10	0.15 ± 0.13
TT58	D22	PT58	100.00 ± 0.00	100.00 ± 0.00	100.00 ± 0.00	89.84 ± 10.16	96.33 ± 6.35	96.30 ± 6.40
TT59	D38	PT59	100.00 ± 0.00	100.00 ± 0.00	100.00 ± 0.00	100.00 ± 0.00	100.00 ± 0.00	100.00 ± 0.00
TT60	D52	PT60	100.00 ± 0.00	100.00 ± 0.00	100.00 ± 0.00	96.61 ± 5.87	96.33 ± 6.35	96.30 ± 6.40
TT61	D34	PT61	100.00 ± 0.00	100.00 ± 0.00	100.00 ± 0.00	96.61 ± 5.87	100.00 ± 0.00	100.00 ± 0.00
TT62	D53	PT62	100.00 ± 0.00	100.00 ± 0.00	100.00 ± 0.00	100.00 ± 0.00	100.00 ± 0.00	100.00 ± 0.00
TT12 (positive control)	D22	PT17	100.00 ± 0.00	100.00 ± 0.00	100.00 ± 0.00	79.30 ± 10.35	96.33 ± 6.35	96.14 ± 6.68
TT13 (positive control)	D23	PT22	96.55 ± 5.98	100.00 ± 0.00	100.00 ± 0.00	50.17 ± 3.35	81.33 ± 12.42	80.29 ± 13.01
Tris buffer (negative control)	-	-	1.00 ± 3.05	4.67 ± 5.71	9.27 ± 7.31	1.00 ± 3.05	4.67 ± 5.71	9.27 ± 7.31

^1^ All data were expressed as mean ± standard deviation (*n* = 3); ^2^ ND: Not determined.

### 3.2. PFGE and RAPD Patterns

When chromosomal DNA of the 60 *Bt* strains (TT1–TT62 except TT23 and TT41) were cut with *Not*I and subjected to PFGE analysis, a total of 54 PFGE patterns were found. Only 4 patterns were shared by two or more strains. For RAPD using primer OPF-06, 41 patterns were found. Only 7 patterns were shared by two or more strains (Table 2). Strains with the same RAPD pattern could be further discriminated by PFGE typing, and vice versa. For example, strains of TT25, TT29, TT42, and TT61, within RAPD pattern of D34, could be further differentiated by PFGE typing; strains in PFGE pattern PT50, *i.e.*, strains TT44 and TT45, could be further discriminated by RAPD typing (Table 2). For the combined use of PFGE and RAPD methods, only four strains, *i.e.*, strains TT47–TT50, were found in a combined pattern, *i.e.*, D43-PT52. Thus, both PFGE and RAPD results demonstrated that there is considerable genetic diversity among the *Bt* strains isolated in Taiwan.

Strains with individual combined patterns showed distinct insecticidal activities; strains that showed the same combined patterns of PFGE and RAPD exhibited similar levels of insecticidal activity. For example, the mortality rates of four strains, *i.e.*, TT47–TT50, within the combined pattern of D43-PT52, were all belonging to strains of high insecticidal activities. The genotypes of *Bt* strains, if determined by highly discriminatory methods, may allow us to identify the newly isolated strains with potential for pesticide use and those strains which have been used in bioinsecticides.

### 3.3. Correlation among Insecticidal Activity, cry Genes, and the Expression Levels of cry1 Genes

A collection of randomly selected 29 *Bt* strains, which exhibited various insecticidal activities, was screened by PCR for the presence or absence of 13 selected *cry* genes, including those associated with the insecticidal activity against *P. xylostella* and those reported by Gaviria Rivera and Priest [26] and Ben-Dov *et al.* [22]. These *cry* genes are widely distributed among strains. All the 29 strains were positive for *cry1* genes; none of the 29 strains carried *cry1E*, *cry3A*, *cry4A2*, *cry8*, *cry9A*, *cry9C* and *cry32Aa* genes. Variation of the presence of *cry1B*, *cry2Aa1*, *cry7*, *cry9Ea*, and *cry22* genes was found among strains (Table 3). Thus, these 5 *cry* genes of *Bt* strains were used to find the correlation with the insecticidal activity against *P. xylostella*.

The correlation between the presence or the absence of these 5 *cry* genes and insecticidal activity of *Bt* strains at the global Cry protein concentration of 25 mg/L was determined using a multiple regression analysis. The presence (1) or absence (0) of these 5 *cry* genes were used as the explanatory variables. Statistical analysis revealed that the presence of *cry2Aa1* and *cry9Ea* genes was positively correlated with the insecticidal activity of *Bt* strains (*p <* 0.05, Table 4). Based on the standardized regression coefficients, the presence of *cry2Aa1* has a greater effect on insecticidal activity of *Bt* strains than other *cry* genes, *i.e.*, *cry1B*, *cry7*, *cry9Ea*, and *cry22* genes. Thus, the presence of *cry2Aa1* might be useful as a reference marker to predict the insecticidal activities of *Bt* strains.

**Table 3:** Comparison of *cry* gene profiles and insecticidal activity of *B. thuringiensis* strains.

Strain	Mortality (%) ^1^			*cry* genes ^2^												
	25 mg/L (global Cry protein)															
	24 h	48 h	72 h	*1*	*1B*	*1E*	*2Aa1*	*3A*	*4A2*	*7*	*8*	*9A*	*9C*	*9Ea*	*22*	*32Aa*
TT59	100.00 ± 0.00	100.00 ± 0.00	100.00 ± 0.00	+	-	-	+	-	-	-	-	-	-	-	-	-
TT62	100.00 ± 0.00	100.00 ± 0.00	100.00 ± 0.00	+	-	-	+	-	-	-	-	-	-	-	-	-
TT61	96.61 ± 5.87	100.00 ± 0.00	100.00 ± 0.00	+	-	-	+	-	-	-	-	-	-	-	-	-
TT5	96.55 ± 5.98	100.00 ± 0.00	100.00 ± 0.00	+	-	-	-	-	-	-	-	-	-	+	-	-
TT27	93.33 ± 5.77	100.00 ± 0.00	100.00 ± 0.00	+	-	-	+	-	-	-	-	-	-	-	-	-
TT29	93.33 ± 5.77	100.00 ± 0.00	100.00 ± 0.00	+	-	-	+	-	-	-	-	-	-	-	-	-
TT30	93.33 ± 11.55	100.00 ± 0.00	100.00 ± 0.00	+	-	-	-	-	-	-	-	-	-	+	-	-
TT25	93.10 ± 11.95	96.33 ± 6.35	96.14 ± 6.68	+	-	-	+	-	-	-	-	-	-	-	-	-
TT50	83.33 ± 15.28	100.00 ± 0.00	100.00 ± 0.00	+	+	-	+	-	-	-	-	-	-	-	-	-
TT49	80.00 ± 10.00	96.67 ± 5.77	96.01 ± 6.91	+	+	-	+	-	-	-	-	-	-	-	-	-
TT17	79.30 ± 10.35	100.00 ± 0.00	100.00 ± 0.00	+	-	-	+	-	-	-	-	-	-	-	-	-
TT12	79.30 ± 10.35	96.33 ± 6.35	96.14 ± 6.68	+	-	-	+	-	-	-	-	-	-	-	-	-
TT47	74.53 ± 9.47	84.00 ± 10.39	83.42 ± 10.50	+	+	-	+	-	-	-	-	-	-	-	-	-
TT34	73.33 ± 5.77	100.00 ± 0.00	100.00 ± 0.00	+	-	-	+	-	-	-	-	-	-	-	-	-
TT48	60.00 ± 10.00	82.67 ± 6.35	88.04 ± 11.96	+	+	-	+	-	-	-	-	-	-	-	-	-
TT41	6.67 ± 5.77	6.93 ± 0.00	0.00 ± 0.00	+	-	-	+	-	-	-	-	-	-	-	-	-
TT19	4.93 ± 4.31	17.01 ± 5.74	24.54 ± 9.62	+	-	-	-	-	-	-	-	-	-	-	-	-
TT2	4.55 ± 3.94	5.28 ± 6.31	4.94 ± 4.28	+	-	-	-	-	-	-	-	-	-	-	-	-
TT16	4.55 ± 3.94	5.67 ± 6.35	2.47 ± 4.28	+	-	-	-	-	-	-	-	-	-	-	-	-
TT1	4.24 ± 3.71	11.91 ± 1.14	10.19 ± 4.81	+	-	-	+	-	-	-	-	-	-	-	-	-
TT44	3.33 ± 5.77	20.72 ± 5.97	12.28 ± 6.91	+	-	-	-	-	-	-	-	-	-	+	-	-
TT24	3.33 ± 5.77	10.38 ± 5.97	13.82 ± 5.97	+	-	-	+	-	-	-	-	-	-	-	-	-
TT4	3.33 ± 5.77	2.33 ± 4.04	4.62 ± 4.00	+	-	-	-	-	-	-	-	-	-	-	-	-
TT23	3.33 ± 5.77	10.07 ± 6.26	20.10 ± 6.57	+	-	-	-	-	-	-	-	-	-	-	+	-
TT57	2.85 ± 4.93	3.58 ± 3.10	0.15 ± 0.13	+	-	-	-	-	-	+	-	-	-	-	-	-
TT11	2.28 ± 3.94	2.00 ± 0.00	0.00 ± 0.00	+	-	-	-	-	-	-	-	-	-	-	-	-
TT43	0.00 ± 0.00	4.62 ± 4.00	2.87 ± 2.49	+	-	-	-	-	-	-	-	-	-	-	-	-
TT56	0.00 ± 0.00	0.00 ± 0.00	0.07 ± 0.13	+	-	-	-	-	-	+	-	-	-	-	-	-
TT3	0.00 ± 0.00	0.00 ± 0.00	0.00 ± 0.00	+	-	-	-	-	-	-	-	-	-	-	-	-
Total				29+ ^3^	4+	0+	16+	0+	0+	2+	0+	0+	0+	3+	1+	0+

^1^ All data were expressed as mean ± standard deviation (*n* = 3); ^2^ The presence (+) or the absence (-) of the specific *cry* gene by PCR was shown; ^3^ Number of strains positive for specific *cry* gene.

**Table 4:** Multiple regression analysis of *cry* genes on insecticidal activity of *B. thuringiensis* strains. *df*, degree of freedom; Sig. *F* change, significance for the change in *R*-square for *F*-test.

Variables	Parameters								
	24 h			48 h			72 h		
	Standardized coefficient	*t*	*p*	Standardized coefficient	*t*	*p*	Standardized coefficient	*t*	*p*
*cry1B*	0.048	0.324	0.749	0.107	0.773	0.447	0.117	0.822	0.419
*cry2Aa1*	0.770	4.369	0.000	0.786	4.794	0.000	0.770	4.551	0.000
*cry7*	−0.008	−0.054	0.957	−0.019	−0.138	0.891	−0.031	−0.211	0.835
*cry9Ea*	0.442	2.822	0.010	0.459	3.154	0.004	0.434	2.888	0.008
*cry22*	0.002	0.016	0.988	0.019	0.143	0.888	0.058	0.414	0.683
*R* square	0.559			0.619			0.594		
*df*1	5			5			5		
*df*2	23			23			23		
Sig. *F* Change	0.001			0.000			0.001		

Since all these strains were positive for *cry1* genes, the expression levels of *cry1* genes were assayed (Table 5). There were 5 strains which showed high expression levels of c*ry1* genes (*Ct* values 17.83–22.76); all these 5 strains exhibited high levels of insecticidal activity (mortality rates >60% or 80%, 25 mg/L global Cry protein for 24 or 72 h). Nine strains which showed low expression levels of c*ry1* genes (*Ct* values 38.25–39.98) exhibited low levels of insecticidal activity. However, two strains, *i.e.*, TT47 and TT49, which belonged to the strains with low expression level of *cry1* genes, were found to have high insecticidal activities (mortality rates > 80%). A Mann-Whitney *U* test was used to determine if the *Bt* strains with high expression levels, *i.e.*, low *Ct* values, of *cry1* genes showed higher insecticidal activities than those with low expression levels, *i.e.*, high *Ct* values, of *cry1* genes. For such a test, *Ct* values <25 were recorded as high expression levels, while those >25 were recorded as low expression levels. Statistical analysis revealed that strains with high expression levels of *cry1* genes exhibited significantly higher insecticidal activities against *P. xylostella* than those with low expression levels of *cry1* genes (exact significance < 0.05 at all time points, *df* = 14). The above data implicate that only high expression level of c*ry1* genes plays a key role to determine the insecticidal activity of *Bt* strains against *P. xylostella* larvae.

**Table 5:** Comparison of expression level of *cry1* and insecticidal activity of *B. thuringiensis* strains. All data were expressed as mean ± standard deviation (*n* = 3); *Ct*, cycle threshold.

Strain	Mortality (%)			Expression level of *cry1* genes (*Ct* value)
	25 mg/L (global Cry protein)			
	24 h	48 h	72 h	
TT30	93.33 ± 11.55	100.00 ± 0.00	100.00 ± 0.00	17.83
TT59	100.00 ± 0.00	100.00 ± 0.00	100.00 ± 0.00	18.92
TT48	60.00 ± 10.00	82.67 ± 6.35	88.04 ± 11.96	19.00
TT50	83.33 ± 15.28	100.00 ± 0.00	100.00 ± 0.00	22.73
TT12	79.30 ± 10.35	96.33 ± 6.35	96.14 ± 6.68	22.76
TT57	2.85 ± 4.93	3.58 ± 3.10	0.15 ± 0.13	38.25
TT23	3.33 ± 5.77	10.07 ± 6.26	20.10 ± 6.57	39.59
TT4	3.33 ± 5.77	2.33 ± 4.04	4.62 ± 4.00	39.79
TT56	0.00 ± 0.00	0.00 ± 0.00	0.07 ± 0.13	39.80
TT47	74.53 ± 9.47	84.00 ± 10.39	83.42 ± 10.50	39.86
TT3	0.00 ± 0.00	0.00 ± 0.00	0.00 ± 0.00	39.87
TT11	2.28 ± 3.94	2.00 ± 0.00	0.00 ± 0.00	39.88
TT19	4.93 ± 4.31	17.01 ± 5.74	24.54 ± 9.62	39.91
TT49	80.00 ± 10.00	96.67 ± 5.77	96.01 ± 6.91	39.98

## 4. Discussion

For decades, worldwide screening followed by isolation and characterization of new *Bt* strains have been undertaken to find out strains with high insecticidal activities [27]. Screening the environment for novel *Bt* strains with high insecticidal activity has become one of the strategies for insect resistance management [28]. On the other hand, considerable genetic diversity among *Bt* strains has been reported. The diversity of *Bt* strains also facilitates the isolation of new types of insecticidal genes [29]. In this regard, a number of techniques have been used to discriminate *Bt* isolates. PFGE is generally considered as an accurate and reproducible method for typing clinically relevant bacteria, and has been used for typing *Bt* strains [26,30]. Another typing method, RAPD, which is fast and simple, also has been applied for the discrimination of *Bt* strains [13]. Although RAPD has been successfully used for the differentiation of *Bt* isolates based on their location of origin, further investigation is still needed to understand their insecticidal spectrum [13]. For PFGE, it has been reported that the clonal structure of *Bt* isolates determined by PFGE may not correlate with their *cry* gene content [10], however, different conclusions have been made by Gaviria Rivera and Priest [26].

In this study, both PFGE and RAPD methods were effective and informative in differentiation of *Bt* strains collected in Taiwan. This is evidenced by the fact that these two methods are capable to discriminate most of the *Bt* strains. When these two methods were combined for the analysis of 60 *Bt* strains isolated in Taiwan, only four strains could not be discriminated. Thus, *Bt* strains in Taiwan are highly diversified in their genome patterns. Such highly genetic diversity may be due to high level of heterogeneity within the chromosomal gene organization for strains. Nevertheless, since strains with individual genotypes showed distinct insecticidal activity against *P. xylostella*, the genotyping data may allow us to identify the *Bt* strains, either newly isolated strains or strains which have been used for bioinsecticide production.

In general, early instar larvae were more susceptible to Cry proteins in many insects than later instar larvae. In this regard, the larval instar used in conducting the bioassays should be considered by not only the most susceptible stage, but also with respect to other factors, such as low mortality in negative controls [31]. Even though older larvae are less susceptible to Cry proteins, to assay the insecticidal activity of *Bt* strains, we used the third instar larvae since such larvae may allow us to select the *Bt* strains with high insecticidal activity. In addition, the assay conditions at 25 mg/L of global Cry protein with 24 h incubation time were used to find the strains with high insecticidal activity since under such conditions, the insecticidal activity of different *Bt* strains could be discriminated effectively (Figure 1). Furthermore, since general stress of larvae, such as nutritively unbalanced food for larvae due to unsuitable host plants, may affect larval vulnerability to *Bt* treatment, and leads to high mortality rates in larval populations [32], the factors of general stress were also considered. *P. xylostella* is considered as one of the most destructive insect pests of cabbage, and effects of insecticides on *P. xylostella* have been evaluated with the use of cabbage [33,34]. In this study, the insecticidal activity of *Bt* strains was also evaluated using cabbage, one of the host plants of P. xylostella. Thus, the possibility that general stress of larvae, such as nutritively unbalanced food, affects the mortality, is little. Under such conditions, 19 of the 60 *Bt* strains isolated in Taiwan were found to have higher insecticidal activities than those of the reference strains.

The *cry* gene content of *Bt* strain may be useful for the prediction of its insecticidal potential, and PCR-based identifications of *cry* genes have been developed to help the screening process [4,8,27]. A previous study revealed that *cry1* and *cry2* were the most abundant genes in the *Bt* strains isolated in Taiwan [11]. In this study, all strains were found to be positive for *cry1* genes. Also, most of the strains with high insecticidal activity were positive for *cry2Aa1*. Our results were in agreement with those described by Chen *et al.* [11]. Moreover, we found that *cry2Aa1* gene was positively correlated with the insecticidal activity of *Bt* strains (Table 4). Regarding *cry1* genes, although all *Bt* strains were positive for *cry1* genes, their insecticidal activities varied. Ferrandis *et al.* [8] reported that the insecticidal toxicity could be related to gene content in most cases, however, one strain without *cry1* genes, showed high toxicity against *P. xylostella*. A *cry* gene, detected by PCR, can be interrupted, mutated, or under control by a defective promoter; the corresponding Cry protein may not be present or present at reduced levels, therefore, contributing minimally to the toxicity [8]. The variety in the expression levels of individual *cry* genes also weakens the correlation between *cry* gene content and the toxicity of *Bt* strains [4].

Thus, the expression levels of *cry1* genes in our *Bt* isolates collected in Taiwan were assayed. All *Bt* strains, which showed high expression levels of the *cry1* genes, exhibited high levels of insecticidal activity. However, two strains, *i.e.*, TT47 and TT49, belonging to the same genotypes, as strains TT48 and TT50, also exhibited high insecticidal activities despite of their low expression levels of the *cry1* genes (Table 5). Thus, there might be some other *cry*-type genes active to *P. xylostella* existing in these *Bt* isolates. Furthermore, other factors, such as β-exotoxins, phospholipases, proteases, chitinases and the secreted VIPs (vegetative insecticidal proteins), are possibly involved in the complete pathogenic effect of a strain [4]. As for the insecticidal activity of Cyt toxins, a review from Frankenhuyzen [14] revealed that results of the activity of Cyt1Aa against *P. xylostella* are conflicting, while for Cyt2Aa, it was not tested against *P. xylostella*. The synergistic interactions of Cry toxins also contribute to the toxicity to a specific insect [35]. In this regard, Porcar and Juárez-Pérez [4] have suggested that for different Cry toxins, the expected dose needed to kill 50% of the insects need to be calculated if the relative proportions of these toxins and the individual toxicity of toxins were known. Nevertheless, although Bt strains used in this study were not checked for the presence of VIP and Cyt proteins, as well as the protein concentrations of the five Cry toxins shown in Table 4, based on the results of this study, it is possible that *Bt* strains with high expression levels of *cry1* genes should be the strains with high insecticidal activity against *P. xylostella*.

## 5. Conclusions

In conclusion, for the isolation and characterization of novel *Bt* strains with high insecticidal activity, both the RAPD and PFGE methods are effective and informative in differentiation of *Bt* strains. Concerning the insecticidal activity against *P. xylostella*, a considerable number of the *Bt* strains isolated in Taiwan were found to have high insecticidal activity, as compared to those of the reference strains isolated from imported bioinsecticides. Also, the presence of *cry2Aa1* gene determined by PCR may be used as a reference marker to predict the insecticidal activity, and only high expression level of the *cry1* genes plays a key role to determine the insecticidal activity of *Bt* strains against *P. xylostella*.
